# Soil Nitrogen Status Modifies Rice Root Response to Nematode-Bacteria Interactions in the Rhizosphere

**DOI:** 10.1371/journal.pone.0148021

**Published:** 2016-02-03

**Authors:** Yanhong Cheng, Ying Jiang, Yue Wu, Tracy A. Valentine, Huixin Li

**Affiliations:** 1 College of Resources and Environmental Sciences, Nanjing Agricultural University, Nanjing, 210095, P.R. China; 2 Jiang Xi institute of red soil, NanChang, 330000, P.R. China; 3 College of Resources and Environment, Henan Agricultural University, Zhengzhou, 450000, P.R. China; 4 Soil and Fertilizer Bureau of Shandong Province, Jinan, 250100, P.R. China; 5 Ecological Sciences, The James Hutton Institute, Invergowrie, Dundee, DD2 5DA, United Kingdom; Chinese Academy of Sciences, CHINA

## Abstract

It has been hypothesized that faunal activity in the rhizosphere influences root growth via an auxin-dependent pathway. In this study, two methods were used to adjust nematode and bacterial populations within experimental soils. One is “exclusion”, where soil mixed with pig manure was placed in two bags with different mesh sizes (1mm and 5μm diameter), and then surrounded by an outer layer of unamended soil resulting in soil with a greater populations of bacterial-feeding nematodes (1mm) and a control treatment (5μm). The second method is “inoculation”, whereby autoclaved soil was inoculated with bacteria (*E*. *coli* and *Pseudomonas*) and Nematodes (*Cephalobus* and *C*. *elegans*). In order to detect the changes in the rice’s perception of auxin under different nutrient and auxin conditions in the presence of soil bacterial-feeding nematodes, responses of soil chemistry (NH_4_^+^, NO_3_^-^ and indole acetic acid (IAA)), rice root growth and the expression of an auxin responsive gene GH3-2 were measured. Results showed that, under low soil nutrient conditions (exclusion), low NO_3_^-^ correlated with increased root branching and IAA correlated with increased root elongation and GH3-2 expression. However, under high soil nutrient conditions (inoculation), a high NH_4_^+^ to NO_3_^-^ ratio promoted an increase in root surface area and there was an additional influence of NH_4_^+^ and NO_3_^-^ on GH3-2 expression. Thus it was concluded that soil bacterial-feeding nematodes influenced soil nutritional status and soil IAA content, promoting root growth via an auxin dependent pathway that was offset by soil nitrogen status.

## Introduction

Root system architecture is an important morphological feature and contributes to plant productivity [1]. The growth and development of plant root systems is not only controlled by the availability and distribution of nutrients within the soil but also regulated by the intrinsic genetic programming of plant [2,3]. Fundamental to these processes are the plant-growth regulating hormones (for example, indole acetic acid, IAA) which regulate aspects of cell elongation, cell division, root initiation and lateral root development, by altering the expression of diverse plant genes [4].

Biotic components of soil also influence the architecture of the plant root system. For example bacterial-feeding nematodes, one of the primary grazers of soil bacteria, have been shown to affect root growth in several plant species, such as sugarcane [5], tomatoes [6] and *Arabidopsis thaliana* [7].

Nematodes’ grazing on bacteria can accelerate bacterial turnover and increase the turnover of soil organic matter [8,9]. This process releases considerable amounts of nitrogen, mainly as ammonium, and thus may enhance plant root growth by a nutritionally based mechanism [10,11]. However, this mechanism does not solely explain the bacterial-feeding nematode stimulation of plant growth [6,12]. Alternatively, nematodes’ grazing on bacteria has been shown to alter microbial community structure [13,14], in the same way as grazing by protozoa [15]. Bonkowski and Brandt have shown that protozoan’ grazing stimulated the proportion of bacteria that produce the plant growth hormone-indole acetic acid (IAA) [16]. In their experiment, the development of a highly branched root system of watercress could be linked to the secretion of this phytohormone [17,18]. Tomato seedlings also developed a more highly-branched root system with longer and thinner roots in the presence of nematodes and this was associated with variation in soil microbial community structure and increased soil auxin content [6]. While effects similar to those of protozoa on root development have been described for the action of bacterial-feeding nematodes in the rhizosphere, the empirical evidence for a hormonal and/or nutrition response of the plant to the rhizosphere microbial loop has remained scarce.

Plant roots are both sources and receptors of molecular signals important for mutualistic bacteria and perhaps other soil organisms, with several trophic groups interacting to influence plant growth, and rhizosphere populations. Phillips [19] introduced the concept of molecular control points in the rhizosphere, in which there is genetic control over responses to signal molecules that have effects at a range of trophic levels. To demonstrate that this concept is applicable to the modified rhizosphere microbial loop, a genetic response in the plant would have to be observed. Auxins regulate a complex signal network to direct plant development and so regulate the expression of many genes [20]. GH3-2 in rice, for example, encodes an IAA-amino synthetase which suppresses the action of IAA by conjugating excess IAA to amino acids [21]. Furthermore, an *Arabidopsis* mutant that over expresses GH3-2 (ydk1-D) displays reduced primary root length and reduced lateral root number [22], suggesting that IAA conjugation and GH3/YDK proteins play an important role in auxin related growth responses by reducing free auxin levels. The GH3-2 gene product also suppresses expansin genes potentially affecting cell expansion [23].

In this study, we used “exclusion” and “inoculation” methods to adjust the nematode and bacterial populations within soils. Through detecting changes in the expression of the auxin responsive gene GH3-2 of rice, we monitored a genetic response of rice as a result of increased nematode activity in the rhizosphere. Meanwhile, the effects of these treatments on soil nutrient (NH_4_^+^, NO_3_^-^) and IAA levels, rice root architecture (root length, no. of root tips, root diameter and root surface area) were analyzed. We present evidence for two complementary pathways involving nematode-bacteria interactions that influence the rate of root growth and root architecture of the rice.

## Materials and Methods

No specific permissions were required for the described field studies. We confirmed that the location of the soil collection was not privately owned or protected in any way, and the field studies did not involve endangered or protected species.

### Nematode and bacteria cultures

Two bacterial-feeding nematode species were used: *Cephalobus* sp. (N1) isolated from the experimental soil; and *Caenorhabditis elegans* (N2). Nematodes were cultured in Petri dishes of nematode growth medium (NGM) inoculated with *Escherichia coli OP50* at 24°C for 10 days [24], and then washed from the plates using sterile water and concentrated by centrifugation (2000 g, 5min) [25]. The obtained nematodes were surface sterilized in 1.0 g L^-1^ streptomycin and 0.02 g L^-1^ cycloheximide for 20 min, and washed five times with sterile distilled water.

Two IAA-producing bacterial species *Pseudomonas* sp. (P) and *Burkholderia* sp. (B) were selected [26], and a non IAA-producing bacterium *Escherichia coli OP50* (E) was chosen as control. Bacteria were grown in nutrient broth (10g L^-1^ peptone, 5 g L^-1^ yeast extract, 10 g L^-1^ NaCl) at 37°C with shaking of 150 rpm for 48 h. After incubation, the culture medium was centrifuged (5000 g, 5 min), and the pellets were washed twice with sterile distilled water. Finally the bacteria were resuspended in sterile distilled water to give a density of 10^7^ colony forming units (cfu) ml^-1^.

### Soil preparation

Alluvial sandy-loam soil from Banqiao town, Nanjing city, Jiangsu Province, China, was sieved(1 mm mesh size)to remove stones, macrofauna and discernible plant residues. Soil characteristics after sieving were pH (H_2_O) 6.8, total organic C 9.20 g kg^-1^, total N 0.89 g kg^-1^, total organic N 9.23 mg.g^-1^.

Soil nematode abundance in the experimental soils was adjusted using two different methods.

1. method for the enrichment of the soil with nematodes: In the exclusion experiment, the selective mesh technique described by Mao was used (S1 Fig) [6]. Briefly, 400 g of field soil mixed with 14 g of pig manure in nylon bags of 5 μm or 1mm pore diameter mesh (inner soil) were placed in a pot and surrounded by 650 g of unamended (outer) soil. Bacterial-feeding nematodes growing in the inner soil were able to migrate through the 1mm diameter mesh to the outer soil, thus giving a greater abundance of bacterial-feeding nematodes in the outer soil surrounding the 1mm diameter mesh than in soil surrounding the 5 μm diameter mesh bag. The 5 μm diameter mesh prevented the movement of nematodes but allowed the diffusion of nutrients into the outer soil. Soil moisture content was adjusted to 60% of field capacity (24.3%, w/w) and three replicate pots of each mesh size were incubated at 22°C in the dark. After four weeks, nematodes were extracted from a sub-sample of the outer soil, counted and preserved [27]. The remaining outer soils were pooled to give soil from around the 1mm mesh bag and soil from around the 5μm mesh bag, and then they were used for the exclusion experiments.
2. soil preparation for the inoculation experiment: For the inoculated experiment, samples of the alluvial sandy-loam soil were autoclaved at 121°C for 2 h on two consecutive days to eliminate resident microorganisms and indigenous nematodes according to the method of [28]. The pH, organic carbon and total N of the soil after sterilization were pH (H_2_O) 7.2, 21.86 g kg^-1^ and 0.93 g kg^-1^, respectively.

### Plant material

Rice seeds (*Oryza sativa* cv. *Japonica*) were surface-sterilized for 20 min in a freshly prepared solution of 1% sodium hypochlorite followed by four washes in sterile distilled water (250 ml wash^-1^). Subsequently, the seeds were incubated at 4°C for 2 days then three days at 22°C in the dark, to synchronize germination.

### Experimental design

Exclusion experiment: For each treatment, 27 replicate pots (containing either 50 g of outer soil from the 1 mm mesh bag or 50 g of outer soil from the 5μm mesh bag), were planted with three rice seedlings each. Before plant incubation, three further samples of each soil type were taken for soil analysis. Pots with plants were incubated in a growth chamber maintained 16 h light at 26°C and 8 h dark at 22°C per day. After 10, 20 and 30 days growth, pots were sampled for root morphology analysis; for root RNA extraction and for soil measurement (IAA, inorganic nitrogen and nematodes).

Inoculation experiment: Pots containing sterilized soil were rewetted to 60% of its water holding capacity (24.3% water content) and then we used sterile water for soil rewetting. The soil was inoculated with bacteria at 10^6^ cfu g^−1^ dry soil and then two days later with nematodes (20.2 ± 0.3 individual g^−1^ dry soil). The experiment consisted of twelve treatments:

Nematodes only: (1) *Cephalobus* (N1); (2) *C*. *elegans* (N2);

*Pseudomonas* bacteria series (IAA-producing): (3) *Pseudomonas* (P); (4) *Pseudomonas* and *Cephalobus* (PN1); (5) *Pseudomonas* and *C*. *elegans* (PN2);

*Burkholderia* bacteria series: (6) *Burkholderia* (B); (7) *Burkholderia* and *Cephalobus* (BN1); (8) *Burkholderia* and *C*. *elegans* (BN2);

*E*. *coli* bacteria series (non IAA-producing): (9) *E*. *coli* (E); (10) *E*. *coli* and *Cephalobus* (EN1); (11) *E*. *coli* and *C*. *elegans* (EN2);

Control—(12) sterilized soil as an uninoculated control (CK).

Soil water content after inoculation of both bacteria and nematodes was maintained 60% water holding capacity by weight. For each treatment, nine replicate pots were planted, each with three germinated rice seedlings planted under aseptic conditions one day after the nematodes were inoculated. Plants were incubated in a growth chamber maintained 16 h light at 26°C and 8 h dark at 22°C per day. Sterile water was added at intervals of one or two days through a sterile tube inserted in the middle of the pot sealed by a sterile film to prevent contamination. On day 0, three replicates of each treatment were sampled for soil analysis. After 14 and 20 days growth, three rice replicates of each treatment were sampled for root morphology and RNA quantification and three replicates for soil measurement.

### Soil analysis

Nematodes were extracted from 20 g fresh soil with a modified Baermann method using trays [29] for 48 h and counted under a dissecting microscope.

NH_4_^+^ and NO_3_^-^ were extracted with 2 M KCl (Soil: KCl; 1: 5) after 30 min shaking (Bremner 1965) and determined using a continuous flow auto-analyser (AA3, Germany). Soil IAA content was measured using high performance liquid chromatography (HPLC) [30]. Soil moisture was measured gravimetrically by drying to constant weight at 105°C for 24h.

### Root analysis

Plant roots were extracted by carefully washing away the soil from the roots using tap water. Images of individual washed roots were acquired using a scanner (LA1600+ scanner, Canada) and root architecture parameters (total length, root tip number, average diameter, total root surface area) were obtained using WinRhizo software (Winrhizo2003b, Canada).

GH3-2 gene was extracted for RNA expression levels analysis. Rice roots were flash frozen in liquid N_2_. Total root RNA was extracted using RNAiso Plus (TaKaRa, Japan) according to the manufacturer’s instructions, followed by RQ1 RNase-Free DNase (Promega, USA) treatment to remove any genomic DNA contamination. First-strand cDNA was synthesized by reverse transcribing 250 ng of total RNA in a final reaction volume of 10 μl using PrimeScript 1st Stand cDNA Synthesis kit (TaKaRa, Japan) according to the manufacturer’s instructions.

The relative abundance of the GH3-2 gene was analyzed. Real-time PCR (qPCR) was performed in an iCycler iQ with the thermal cycling system (BIO-RAD, USA), using SYBR Premix Ex Taq (2x) (TakaRa, Japan) according to the manufacturer’s instruction. Control qPCRs with no template were also performed for each primer pair (Table 1). All the qPCRs were performed under the following conditions: one cycle of 30 s at 95°C, and 40 cycles of 5 s at 95°C, 60 s at 60°C and one cycle of 15 s at 95°C, 30 s at 60°C in 8-well optical reaction tubes (Axygen, USA) using the primer sequences shown in Table 1. Three biological replicates for each treatment were used for real-time qPCR analysis, with two technical replicates. The relative abundance of the GH3-2 gene was calculated using Excel (Microsoft) with respect to the UBQ5 gene for normalization purposes.

**Table 1:** Primer sequences for relative real-time PCR quantification of GH3-2 expression.

Gene name	Accession no.	Primer sequences	Reference
UBQ5	EF47023	5’-ACCACTTCGACCGCCACTACT-3’ 5’-ACGCCTAAGCCTGCTGGTT-3’	Jain et al., 2006
GH3-2	LOC_Os01g55940	5’-AGAGGGCATCAGGGCCATAC-3’ 5’-CTACCACTGGACAGCATGATCTAAG-3’	Jain et al., 2006

### Statistical analysis

Root and soil parameters were analyzed by ANOVA with bacteria, nematode and sampling day as fixed factors. Comparisons between means were performed at the 5% probability level with a Fisher’s least significant difference (LSD) test (*P* < 0.05) using the statistical package SPSS16.0 or using Unbalanced ANOVA (GENSTAT v13). Restricted Maximum Likelihood (REML) analysis was applied to soil and root parameters to assess whether increased soil faunal abundance stimulates root growth via an IAA-dependent pathway or via nutritional effects (GENSTAT v13), with “individual pot” included as a random parameter term in all models.

## Results

In the results below, we compared the effects of two different methodologies (exclusion and inoculation) for manipulating the soil IAA- producing bacteria on each of the necessary parameter relationships described above.

### Exclusion experiment

#### Soil parameters

**Fig 1:**
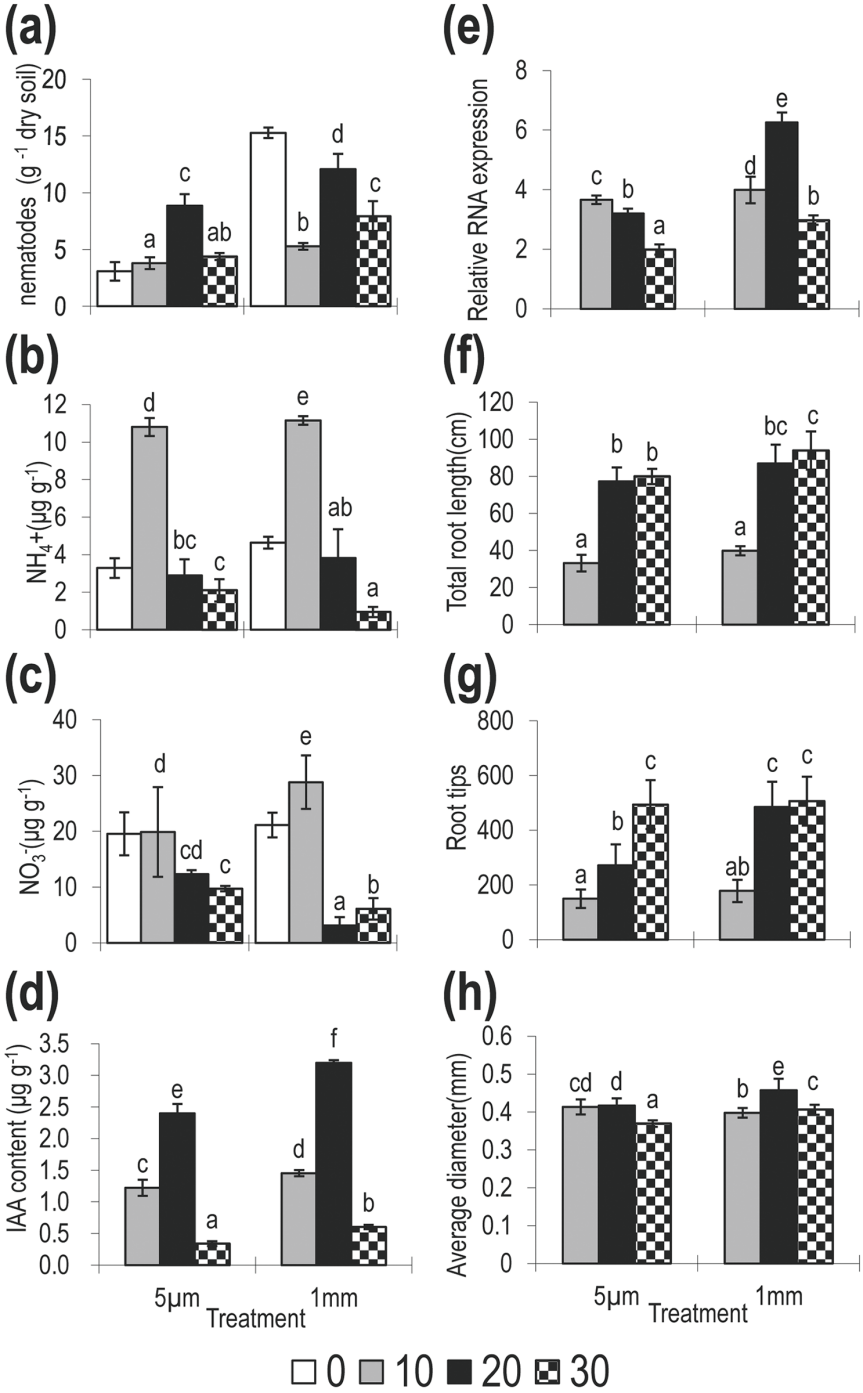
Dynamic of soil parameters, root architecture and relative GH3-2 expression in rice roots in the ‘exclusion’ experiment, after 0, 10, 20 and 30 days of plant growth. Comparisons are shown for two soil types treated using either a 1mm or 5 μm, to allow or exclude (respectively) bacterial-feeding nematodes and where: (a) nematode abundance (b): NH_4_^+^, (c) NO_3_^-^,(d) IAA content, (e) relative GH3-2 expression: (f) total root length; (g) root tips; (h) root average diameter.

Nematode abundance in the 1 mm treatment were significantly greater than in the 5μm treatment throughout the experiment (Fig 1a ANOVA *P*<0.001, F = 34.10), although the nematode abundance varied significantly over time (ANOVA *P*<0.001, F = 56.24). There was also a significant increase in the percentage of bacterial-feeders in the 1mm mesh treatment to 95% of the nematode community (*P*<0.05, F = 56.63), but no such change in the 5μm mesh treatment.

Soil mineral N levels increased from day 0 to day 10 after planting and during this period were significantly greater in the 1mm compared to the 5μm treatment (Fig 1b and 1c). An accumulation of NH_4_^+^ after 10 days in both treatments and a significantly larger increase in NO_3_^-^ concentration in the 1mm treatment (Fig 1b and 1c) were found. Mineral N concentrations then decreased sharply from day 10 to day 30 (Fig 1b and 1c) and during this period the NO_3_^-^ concentration was lower in the 1mm treatment than in the 5μm treatment (Fig 1b and 1c, *P =* 0.006, <0.001 F = 9.14, 27.06 respectively (Time:Treatment)).

REML analysis of the effect of nematode abundance on NH_4_^+^ / NO_3_^-^ concentrations suggested a significant effect of NO_3_^-^ (*P =* 0.005,. Both NH_4_^+^ / NO_3_^-^ showed a significant effect of the interaction between the sampling day and nematode abundance (*P =* 0.009 and *P =* 0.033). Only NO_3_^-^ showed a significant positive relationship with nematode abundance as a main effect.(*P =* 0.005, Table 2a).

**Table 2:** REML analysis of the ‘exclusion’ dataset. (a) relationship between the number of nematodes and soil chemistry (IAA, NH_4_^+^, NO_3_^-^). (b) Relationship between soil chemistry and root growth parameters. Individual pots included as random model. Effect = effect(s.e.). Bold means significant interactions.

**(a)**	**DAY**	**No of Nematodes**	**DAY. No of Nematodes**	
**NH**_**4**_^**+**^				
*P*	**<0.001**	0.163	**0.009**	
F	**55.19**	2.21	**7.11**	
**NO**_**3**_^**-**^				
*P*	**<0.001**	**0.005**	**0.033**	
F	**20.96**	**11.56**	**4.61**	
**IAA**				
*P*	**<0.001**	**<0.001**	0.445	
F	**296.87**	**24.66**	0.87	
**(b)**	**DAY**	**IAA**	**NH**_**4**_^**+**^	**NO**_**3**_^**-**^
**Root Length**				
*P*	**<0.001**	**0.021**	0.867	0.061
F	**232.98**	**9.59**	0.03	5.29
**Root Tips**				
*P*	**<0.001**	0.063	0.45	**0.033**
F	**32.56**	5.11	0.65	**7.6**
**Root Diameter**				
*P*	**0.004**	**0.013**	**0.044**	0.345
F	**16.09**	**12.37**	**6.49**	1.05
**Root Surface Area**				
*P*	**<0.001**	0.553	0.862	0.944
F	**27.08**	0.4	0.03	0.01

Note: REML analysis performed on LN (x+1) transformed data, except for Average diameter (-Double log +100), length and Tips (no transformation).

#### Changes in soil IAA content and GH3-2 expression *in planta*

**Fig 2:**
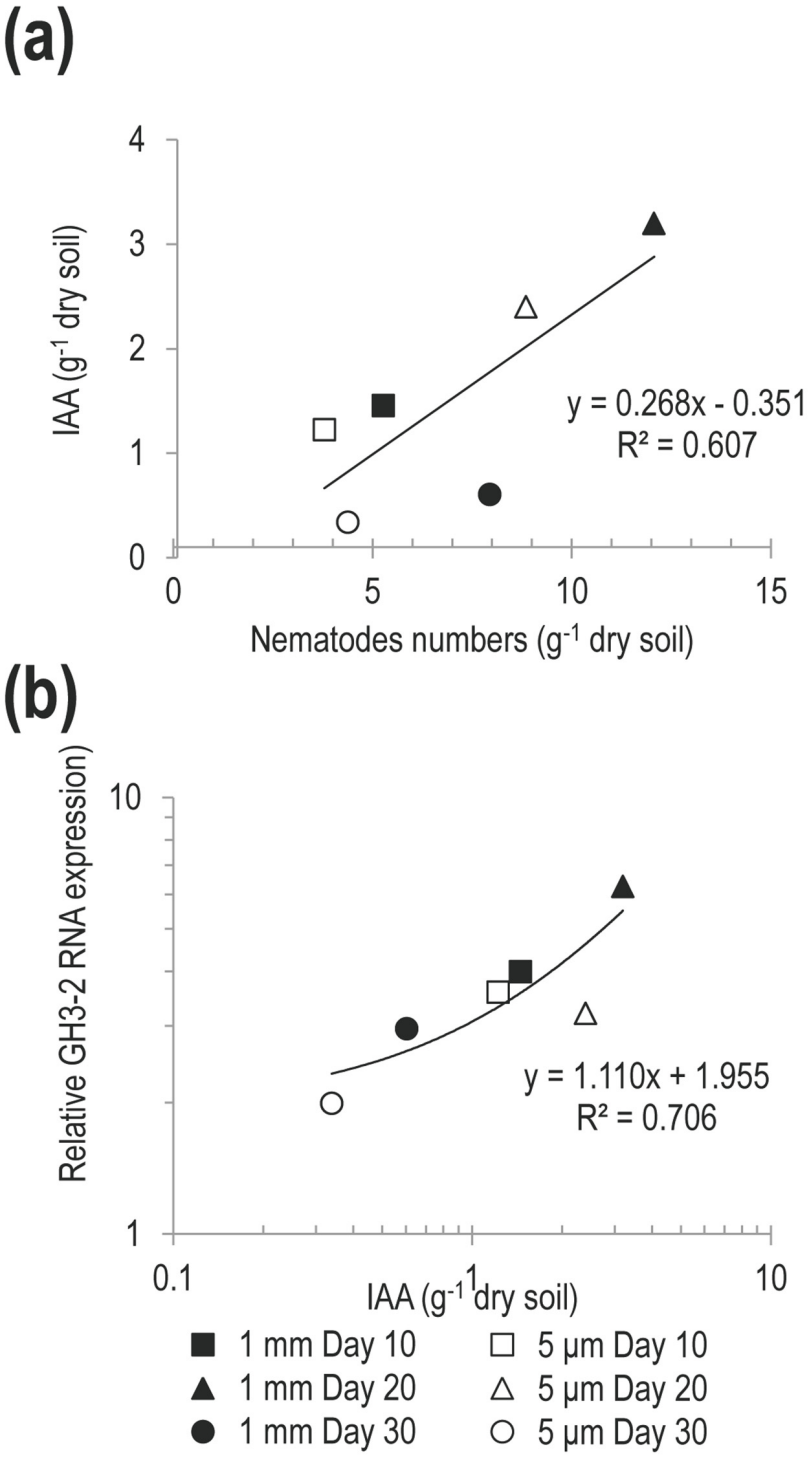
Relationship between soil IAA content and (a) Nematode abundance or (b) relative GH3-2 expression in rice roots. 1mm: soil contains more bacterial-feeding nematodes than 5 μm: see text for details.

Soil IAA concentration was greater in the 1mm treatment than in the 5μm treatment (Fig 1d, *P*<0.001, F = 36.86) and individual pots with higher nematode abundance exhibited higher IAA concentrations (Fig 2a, r^2^ = 0.607). This was supported by REML analysis showing a significant effect of nematode abundance on IAA concentration (Table 2a, *P*<0.001). While IAA concentrations after 30 days are also significantly lower than on days 10 and 20 (*P<*0.001, Table 2a, Fig 1d).

The expression levels of the IAA responsive GH3-2 gene *in planta* were also significantly higher in the 1mm treatment versus 5μm treatment (Fig 1e, ANOVA, *P*<0.001, F = 51.31). They also showed a significant positive correlation with the concentration of IAA in the soil (Fig 2b r^2^ = 0.706, and Table 3, correlation coefficient = 0.841, *P* = 0.0361).

**Table 3:** Correlation statistics between GH3-2 relative expression and soil chemistry or root growth parameters in the ‘exclusion’ experiment. Bold means significant interactions.

Parameter	IAA	NH_4_^+^	NO_3_^-^	No of Nematodes	Root length	Root growth	Root Tips	Average Diameter	Root Surface Area
***P***	**0.036**	0.985	0.599	0.163	0.866	0.064	0.651	**0.015**	0.877
**Correlation coefficient**	**0.841**	0.010	-0.274	0.649	-0.080	0.785	0.237	**0.899**	-0.082

#### Response of root growth to changes in nematode abundance

Roots in the 1 mm (high nematode abundance) treatment were longer, had more root-tips and a larger average diameter than in the 5 μm treatment (Fig 1f–1h, *P*<0.001, F = 25.99, 17.73, 16.71 respectively). Root length was positively correlated to high soil IAA concentration (*P* = 0.021, Table 2b), but was not directly related to NH_4_^+^ (*P* = 0.867) or NO_3_^-^ (*P* = 0.061) concentration. There was some evidence that root growth promotion was related to an interaction between IAA and NH_4_^+^ (*P* = 0.049, data not shown), but there was no strong evidence of a direct relationship between IAA or NH_4_^+^ concentration and tip number (*P* = 0.063 and 0.450 respectively, Table 2b). The increase in the number of root tips was correlated to a decrease in the NO_3_^-^ concentration when IAA and NH_4_^+^ were included in the analysis (*P* = 0.033, Table 2b). However, this link was not significant if IAA and NH_4_^+^ were removed from the analysis. The diameter of the roots increased in line with higher IAA (*P* = 0.013, Table 2b) and GH3-2 expression (correlation coefficient = 0.899, *P* = 0.015, Table 3), but was decreased under higher NH_4_^+^ concentrations (*P* = 0.044, Table 2b). There was no evidence of a relationship between IAA, NO_3_^-^ or NH_4_^+^ and the overall root surface area.

### Inoculation experiment

**Fig 3:**
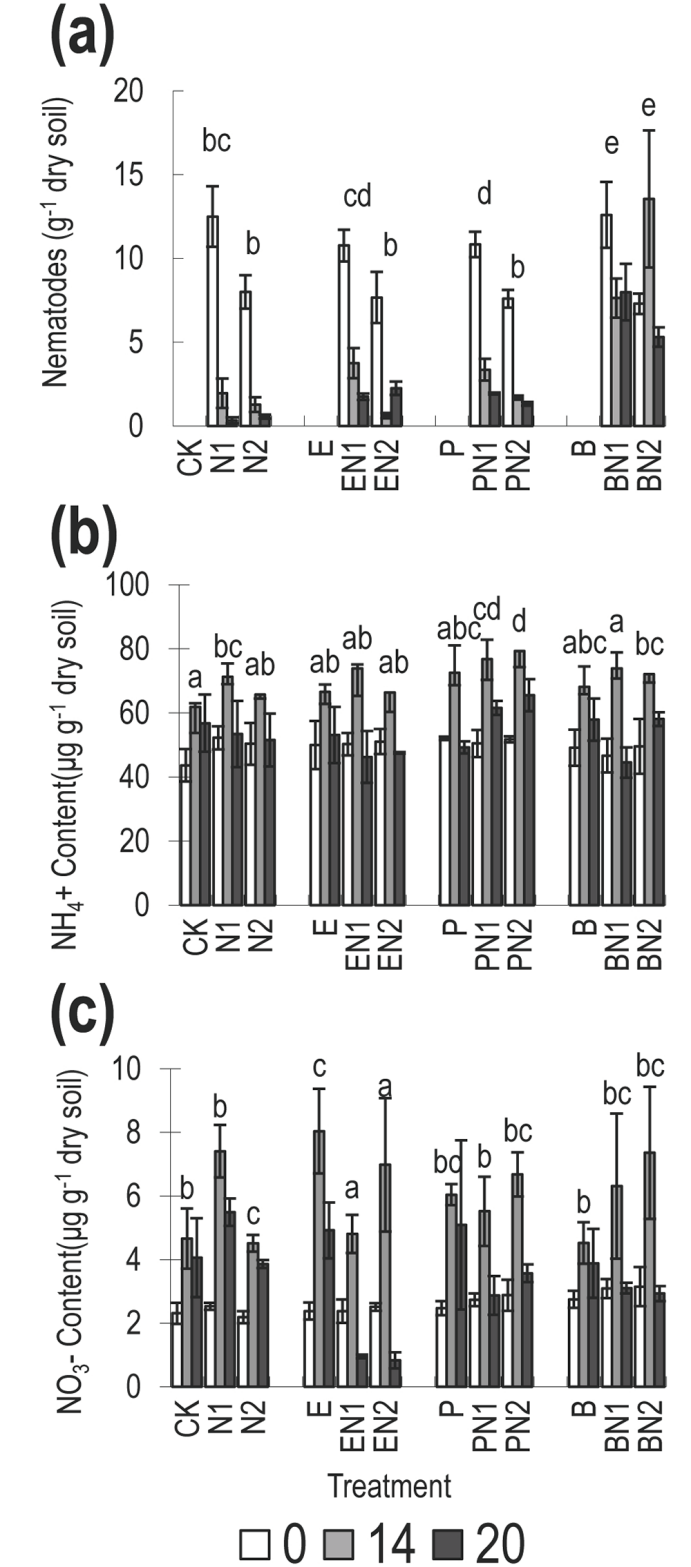
Dynamic of soil mineral nitrogen in the ‘inoculation’ experiment; (a) nematode abundance, (b): NH4+, (c) NO3-, before and 14 and 20 days after inoculation of sterilised soil with: CK = sterilized soil; N1 = *Cephalobus*; N2 = *C*. *elegans*; E = *E*. *coli*; EN1 = *E*.*coli* and *Cephalobus*; EN2 = *E* and N2; P = *Pseudomonas*; PN1 = *P* and N1; PN2 = *P* and N2; B = *Burkholderia*; BN1 = *B* and N1; BN2 = *B* and N2.

**Fig 4:**
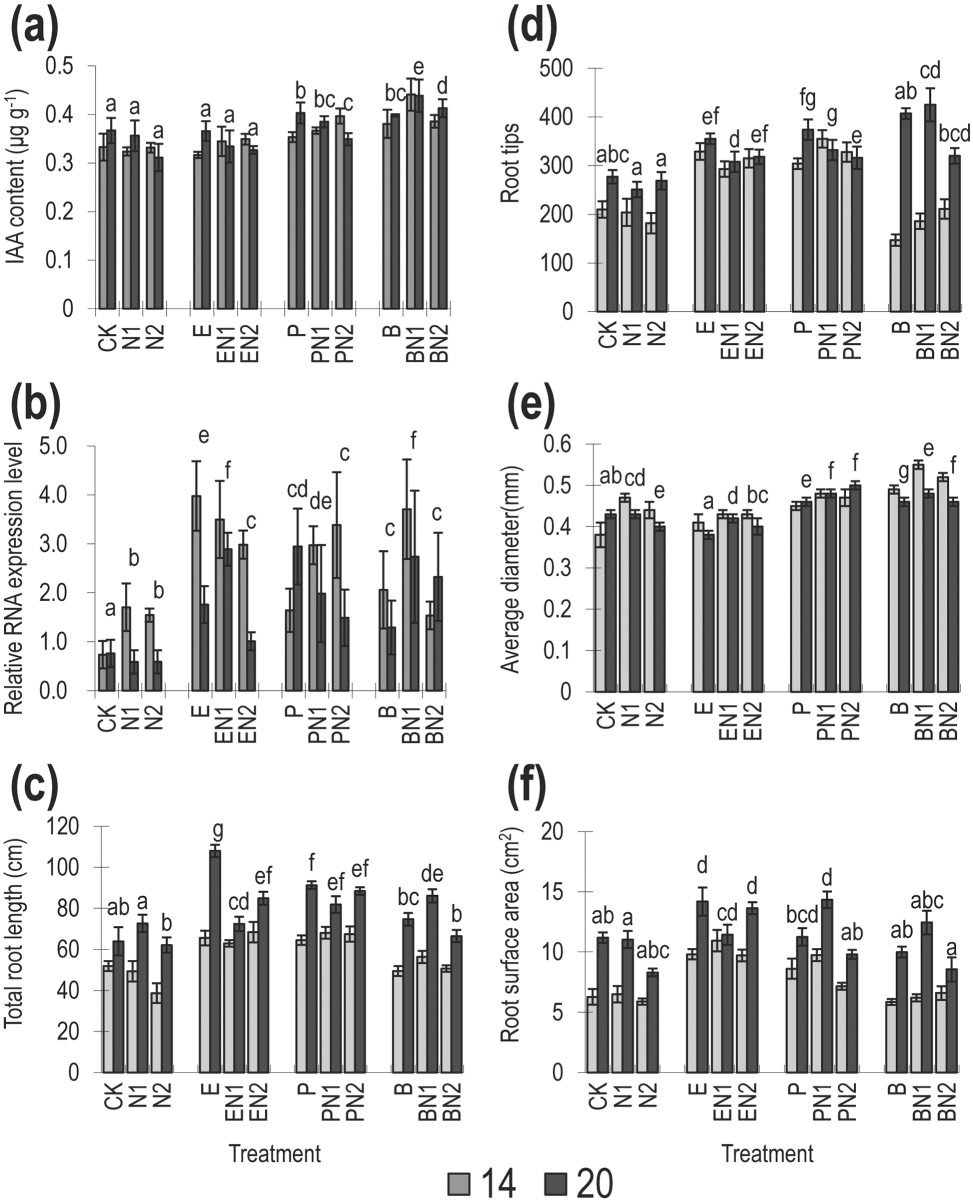
Effects of bacterial-feeding nematodes on soil IAA content, root architecture and and relative GH3-2 expression in rice root in the ‘inoculation’ experiment. (a) IAA content; (b) relative GH3-2 expression, (c) total root length; (d) root tips; (e) root average diameter; (f) root surface area. Treatments are as Fig 3.

The dynamics of the soil and root parameters over the course of the inoculation experiment are shown in Figs 3 and 4. The parameters were analyzed as follows: Effect of bacteria ad nematode abundance on soil parameters (Table 4a); effect of soil parameters on plant root parameters (Table 4b and 4c); effect of bacteria vs no bacteria in combination with or without nematodes on all parameters (Table 5); or more specifically the effect of IAA producing bacteria vs no or non-IAA producing bacteria in combination with or without nematodes on all parameters (Table 6).

**Table 4:** REML analysis of the ‘inoculation’ dataset,(a) relationship between bacteria type, nematode number and soil chemistry. (b) Relationship between soil chemistry, root growth parameters and *in planta* expression of GH3-2. (c) Relationship between soil chemistry and root growth parameters and links between soil chemistry and GH3-2 expression *in planta*. Individual pot was included as random term. Bold means significant interactions.

**(a)**	**DAY**	**Bacteria**	**No of Nematode**	**Bacteria. Nematode**					
**NH**_**4**_^**+**^									
*P*	**<0.001**	**0.004**	0.259	0.173					
F	**96.95**	**4.71**	1.29	1.7					
**NO**_**3**_^**-**^									
*P*	**<0.001**	0.057	0.773	0.331					
F	**54.32**	2.6	0.08	1.15					
**IAA**									
*P*	**0.002**	**<0.001**	**0.012**	0.425					
F	**6.59**	**45.53**	**6.52**	0.94					
**(b)**	**DAY**	**IAA**	**NH**_**4**_^**+**^	**NO**_**3**_^**-**^	**IAA.NH**_**4**_^**+**^	**IAA.NO**_**3**_^**-**^	**NH**_**4**_^**+**^**.NO**_**3**_^**-**^	**IAA.NH**_**4**_^**+**^**.NO**_**3**_**-**	
**Root Length**									
*P*	**<0.001**	0.346	0.752	0.087	0.616	0.193	0.215	0.141	
F	**55.69**	0.9	0.1	3.03	0.25	1.73	1.57	2.23	
**Root Tips**									
*P*	**<0.001**	0.253	0.832	0.446	0.065	0.256	0.117	**0.048**	
F	**26.29**	1.33	0.05	0.59	3.55	1.11	2.54	**4.09**	
**Root Diameter**									
*P*	0.102	**<0.001**	**0.011**	0.676	0.627	0.267	0.78	0.641	
F	2.77	**16.66**	**6.9**	0.18	0.24	1.26	0.08	0.22	
**Root Surface Area**									
*P*	**<0.001**	0.475	0.99	0.69	0.12	0.468	**0.005**	0.123	
F	**70.7**	0.52	0	0.16	2.5	0.53	**8.58**	2.45	
**GH3-2**									
*P*	**0.003**	**0.002**	0.12	0.057	0.09	0.977	**0.001**	0.733	
F	**10.13**	**13.24**	2.55	3.88	3.13	0	**12.08**	0.12	
**(c)**	**DAY**	**IAA**	**NH**_**4**_^**+**^	**NO**_**3**_^**-**^	**IAA.NH**_**4**_^**+**^	**IAA.NO**_**3**_^**-**^	**NH**_**4**_^**+**^**.NO**_**3**_^**-**^	**IAA.NH**_**4**_^**+**^**.NO**_**3**_**-**	**GH3-2**
**Root Length**									
*P*	**<0.001**	0.388	0.284	0.363	0.66	0.072	0.903	0.549	**0.021**
F	**38.28**	0.76	1.17	0.85	0.2	3.43	0.02	0.37	**5.81**
**Root Tips**									
*P*	**0.001**	0.937	0.53	0.664	0.28	0.165	0.498	0.216	**0.044**
F	**12.22**	0.01	0.4	0.19	1.2	2.01	0.47	1.59	**4.37**
**Root Diameter**									
*P*	0.145	**0.035**	0.176	0.958	0.461	0.308	0.857	0.825	0.565
F	2.22	**4.79**	1.91	0	0.56	1.07	0.03	0.05	0.34
**Root Surface Area**									
*P*	**<0.001**	0.295	0.675	0.586	0.206	0.59	0.205	0.745	0.071
F	**43.95**	1.13	0.18	0.3	1.65	0.3	1.67	0.11	3.47

Note: REML analysis performed on -DL(x+100) IAA, otherwise LN (x+1) transformed data except for Length and Tips no transformation necessary.

**Table 5:** REML Effect of bacteria and nematode presence on the soil chemistry and root growth parameters in the ‘inoculation’ experiment. Bold means significant interactions.

	DAY	Bacteria presence	Nematode presence	Bacteria presence.Nemtode presence	
**Nematode numbers**					
*P*	**<0.001**	**0.003**	**<0.001**	0.086	(Y* > N*) (*Y > *N)
F	**10.93**	**9.53**	**65.82**	3.03	
**NH**_**4**_^**+**^					
*P*	**<0.001**	0.305	0.22	0.08	NS
F	**109.86**	1.1	1.59	3.16	
**NO**_**3**_^**-**^					
*P*	**<0.001**	0.252	0.08	**0.03**	NN = YY < YN = NY
F	**35**	1.33	3.15	4.91	
**IAA**					
*P*	0.092	**<0.001**	0.666	0.196	Y* > N*
F	2.47	**19.13**	0.19	1.7	
**Root Length**					
*P*	**<0.001**	**<0.001**	**0.041**	0.248	(Y*>N*)
F	**81.74**	**31.02**	**4.3**	1.36	
**Root Tips**					
*P*	**<0.001**	**<0.001**	0.388	0.547	Y* > N*
F	**33.29**	**28.29**	0.75	0.37	
**Root Average Diameter**					
*P*	0.139	**0.002**	0.12	0.219	Y* > N*
F	2.23	**10.01**	2.46	1.54	
**Root Surface Area**					
*P*	**<0.001**	**0.001**	0.803	0.73	Y* > N*
*F*	**65.63**	**11.68**	0.06	0.12	
**GH3-2**					
*P*	**0.018**	**<0.001**	0.156	0.981	Y* > N*
F	**5.99**	**27.54**	2.07	0	

Note: REML analysis performed on -DL(x+100) IAA, otherwise LN (x+1) transformed data except for Length and Tips no transformation necessary. YY = bacteria present, nematodes present, YN bacteria present, No nematodes, NY No bacteria, Nematodes present, NN No bacteria added, No nematodes added. NS = no significant difference.

* means significant differences between treatments, the same below.

Model = Day + (Bacteria Presence*Nematode Presence)

**Table 6:** REML Effect of IAA-producing bacteria (*Pseudomonas* sp. and *Burkholdeeria* sp.) and nematode presence on the soil chemistry and root growth parameters in the ‘inoculation’ experiment. Bold means significant interact-ions.

	DAY	IAA Bacteria presence	Nematode presence	IAA Bacteria presence.Nematode presence	
**Nematode numbers**					
*P*	**<0.001**	**<0.001**	**<0.001**	**0.002**	YN < NN < NY < YY
F	12.22	**12.15**	**73.39**	**10.81**	
**NH**_**4**_^**+**^					
*P*	**<0.001**	**0.007**	0.205	0.731	Y* > N*
F	**86.66**	**7.93**	1.64	0.12	
**NO**_**3**_^**-**^					
*P*	**<0.001**	0.212	0.084	0.140	NS
F	**34.02.**	1.58	3.06	2.22	
**IAA**					
*P*	**0.014**	**<0.001**	0.490	0.053	YY > NY > NN = YN
F	**4.63**	**93.96**	0.48	3.86	
**Root Length**					
*P*	**<0.001**	0.382	0.056	0.143	NS
F	**60.41**	0.77	3.76	2.18	
**Root Tips**					
*P*	**<0.001**	0.083	0.370	0.310	NS
F	**25.76**	3.08	0.81	1.04	
**Root Average Diameter**					
*P*	**0.039**	**<0.001**	**0.05**	**0.015**	YN = YY > NY > NN
F	**4.38**	**93.53**	**3.95**	**6.18**	
**Root Surface Area**					
*P*	**<0.001**	0.106	0.754	0.499	NS
*F*	**59.1**	2.66	0.1	0.46	
**GH3-2**					
*P*	**0.045**	0.102	0.156	0.994	NS
F	**4.21**	2.77	2.07	0	

Note: REML analysis performed on -DL(x+100) IAA, otherwise LN (x+1) transformed data except for Length and Tips no transformation necessary. YY = IAA bacteria present, nematodes present, YN IAA bacteria present, No nematodes, NY No IAA bacteria, Nematodes present, NN No IAA bacteria added, No nematodes added.

Model = Day + (IAA bacteria Presence*Nematode Presence.

#### Effect of bacterial and nematode inoculations on nematode abundance and soil NH_4_^+^ and NO_3_^-^ concentration

All treatments exhibited a decline in nematode abundance over the course of the experiment (ANOVA *P*<0.001, F = 21.31) except for *C*. *elegans* with either *E*. *coli* (EN2) (lowest at 14 days) or *Burkholderia* (BN2) (peaked at 14 days) (Fig 3a). The nematode abundance also varied significantly between treatments (ANOVA *P*<0.001, F = 20.31) with *Burkholderia* supporting the greatest nematode abundance at day 14 and day 20.

Soil NH_4_^+^ and NO_3_^-^ concentration peaked at day 14 in all treatments (ANOVA *P<*0.001, Fig 3b and 3c). Changes in NH_4_^+^ were associated with difference bacterial treatments (*P =* 0.004 Table 4), particularly IAA bacterial presence rather than bacterial presence per se (contrast *P =* 0.305 Table 5 vs *P =* 0.007 Table 6), There was no evidence of the effect of nematode presence/absence on NH_4_^+^ concentration. NO_3_^-^ concentration was not significantly affected by the nematode numbers x bacteria (Table 4) but there was a significant interaction when data was analyzed as bacterial presence, nematode presence (*P =* 0.03, Table 5) but this was not linked to IAA bacterial presence (*P =* 0.14, Table 6).

#### Changes in soil IAA content and GH3-2 expression *in planta* after bacteria and nematode inoculations

Simple ANOVA analysis suggested effects of bacterial treatments and bacteria x nematode treatment interactions on IAA concentration (ANOVA *P<*0.001, 0.034, F = 67.66, 2.43) respectively. The concentration of IAA was higher in soils with bacteria vs no bacteria (*P<*0.001, Table 5) and for the IAA producing bacteria *Pseudomonas* and *Burkholderia* alone than in soils with no bacteria or *E*. *coli* (Fig 4a, *P<*0.001, Table 6). Higher concentrations of IAA were also correlated to higher nematode abundance (REML *P* = 0.012, Table 4a). There was no evidence of bacterial and nematode interaction on the IAA concentration when analyzed as bacteria treatment x nematode abundance (*P =* 0.425, Table 4), nor bacteria presence x nematode presence (*P =* 0.196, Table 5), nor between IAA bacterial presence/absence x nematode presence (*P =* 0.053, Table 6). Higher concentrations of IAA were found in treatments with both IAA-producing bacteria and nematodes than in non-IAA bacteria, nematode alone and control treatments (Fig 4a). The overall average concentration of IAA in the inoculation experiment was approximately a factor of 10 lower than the concentration found in the exclusion experiment.

GH3-2 expression was greater in the 14 day root samples than in the 20 day samples (Fig 4b, ANOVA *P* = 0.003, F = 9.66), except for *Pseudomonas* alone treatment. There was evidence of treatment effects on the expression of GH3-2, however, the variation was associated with bacteria alone (highest in the presence of *Berkolderia* and *E*. *coli*, ANOVA *P*<0.001, F = 18.09, REML *P*<0.001, Table 5) rather that the IAA producing bacteria (*P =* 0.102, Table 6) or nematodes alone (ANOVA *P* = 0.002, F = 7.35) but this was not supported by the presence/absences REML analysis (*P =* 0.156, 0.156, Tables 5 and 6). Higher GH3-2 expression levels were found to correlate with higher soil IAA content (REML *P* = 0.002, F = 13.24, Table 4b), but not higher NH_4_^+^ or NO_3_^-^ (REML *P* = 0.120, 0.057 respectively, Table 4b). However, there was also an equally significant positive relationship between the NH_4_^+^.NO_3_^-^ interaction term and GH3-2 expression (REML *P* = 0.001, F = 12.08, Table 4b).

#### Response of root growth to changes in faunal composition

Evidence of treatment effects on root parameters due to bacteria composition (ANOVA *P< =* 0.006, F = 7235.77, 108.65, 7.88, 200.41, Root length, tips, diameter, surface area respectively), nematode composition (ANOVA *P =* 0.016, 0.011, F = 4.24, 4.63 Root length, diameter respectively) and due to bacteria-nematode interactions were found (Fig 4c–4f, ANOVA *P*<0.001, F = 8.18, 4.83, 6.40, 5.60, Root length, tips, diameter, surface area respectively), Shorter roots and fewer root-tips were found in the no bacteria and *Burkholderia* samples than in the *E*. *coli* or *Pseudomonas* treatments (Fig 4c–4f, *P<0*.*001*, *F =* 53.15, 53.24 respectively) thus there was no link specifically to the presence or absence of IAA producing bacteria (*P =* 0.382, 0.083, respectively, Table 6). The presence or absence of bacteria was also associated with changes in root diameter and surface area (*P =* 0.002, 0.001 respectively, Table 5) but only root average diameter was linked to IAA producing bacteria presence/absence (*P*<0.001, Table 6) and IAA bacteria presence/absence interaction with nematode presence/ absence (*P =* 0.015, Table 6)

No significant relationship of IAA, NH_4_^+^ or NO_3_^-^ were found with root length, root tip number (Table 4b), except for a significant negative correlation of the triple interaction term (IAA.NH_4_^+^.NO_3_^-^) on tip production (*P* = 0.048, Table 4b). However, an increase in the average diameter of roots was correlated with higher concentrations of soil IAA, and NH_4_^+^ (REML *P*<0.001, 0.011 respectively, Table 4b). There was evidence of an effect on root surface area due to an interaction between NH_4_^+^ and NO_3_^-^ (REML *P* = 0.005, Table 4b), but only when IAA was also included in the analysis.

In the inoculation experiment GH3-2 expression alone was not significantly related to any of the root growth parameters, we therefore used the model DAY+IAA*NH_4_^+^*NO_3_^-^+GH3-2 to investigate the role of GH3-2 in root growth in the inoculation experiment (Table 4b and 4c). Significant links were found between GH3-2 expression and root length (REML *P* = 0.021, Table 4c) and the number of root tips (REML *P* = 0.044, Table 4c). For root tips there was evidence that the GH3-2 may be interacting antagonistically with the effects of the soil chemistry, as the previous analysis not including GH3-2 gave a significant negative link between all three nutrients and root tip numbers (REML *P* = 0.048, Table 4b). Whereas when GH3-2 was included in the analysis, the IAA-NH_4_^+^-NO_3_^-^ term become a non-significant effect (REML *P* = 0.216, Table 4c). Inclusion of GH3-2 in the analysis also reduced the significance of the effect of NH_4_^+^ on root diameter (REML *P* = 0.011 reduced to *P* = 0.176 on inclusion of GH3-2), Table 4b and 4c), and NH_4_^+^.NO_3_^-^ on root surface area (REML *P* = 0.005 reduce to 0.205 on inclusion of GH3-2, Table 4b and 4c).

### Summary interactions

**Fig 5:**
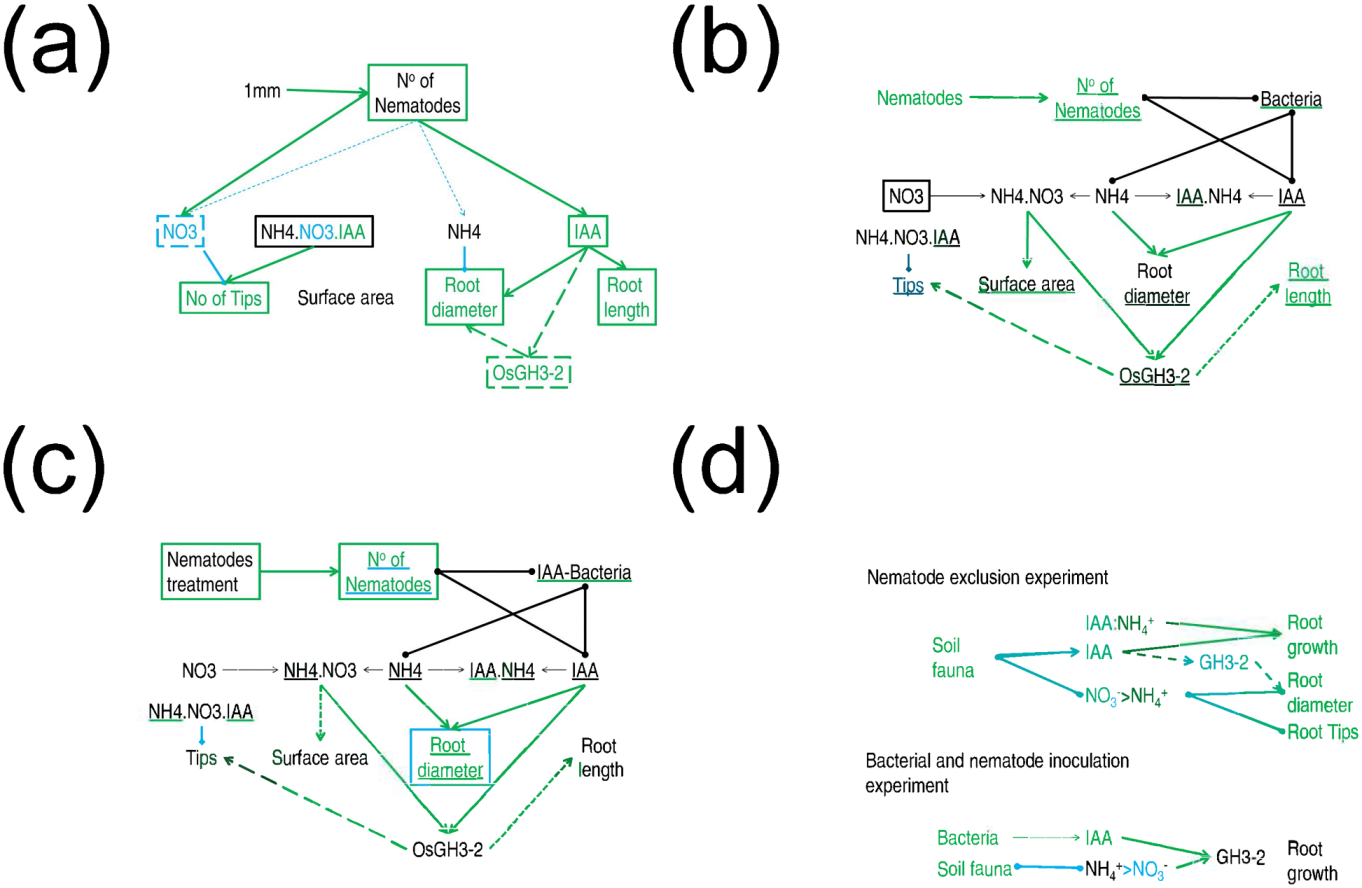
Overview of the effects of changes of faunal composition on soil chemistry and root parameters (a) the ‘exclusion’ dataset—Effect of treatment on parameter as supported by REML (See Table 5—Text and box colour, GREEN 1mm>5mm, BLUE 1mm<5mm, Dashed line only significant in interaction with DAYS). Lines and arrows interactions parameters correlations supported by REML (See–Table 2 lines and arrows—GREEN positive effect, BLUE Negative effect, Pale blue dotted line interaction only significant due to interaction with DAYS). Green dashed line interactions supported by correlations (Fig 3) (b-c) the ‘inoculation’ dataset (b) Effect of the presence of any bacteria and nematodes, (c) Effect of the presence of specifically IAA-producing bacteria (vs other bacteria and no bacteria) and nematodes. (See Table 6) Text colour = nematode effect, Underline colour = bacterial effect, box = significant interaction effect. GREEN = positive effect of the presence, BLUE = negative effect. BLACK = more complex interactions. Lines and arrows interactions parameters correlations supported by REML (See Table 4 lines and arrows—GREEN positive effect, BLUE Negative effect, BLACK significant interactions with either bacteria (but not specifically IAA producing bacteria or nematode). Green dashed line weaker potential interactions (see Table 4d) (d) Comparison of the main effects in the two experiments. GREEN—increased under higher faunal levels or positive interactions BLUE—decreased under higher faunal levels or negative interaction.

Although bacteria and nematode treatments significantly affected soil and root growth parameters in both the inoculation and exclusion experiments, only nematode numbers, NH_4_^+^, IAA and root average diameter changes were associated with IAA bacteria presence specifically and only nematode numbers and root average diameter were affected by the presence of IAA bacteria in the presence of nematodes in the inoculation experiment. A graphical summary of the main effects resulting from the REML analysis in the exclusion experiment (Fig 5a) and the effects of the presence of bacteria (Fig 5b) or specifically IAA-producing-bacteria (Fig 5c) and nematodes in the inoculation experiment are presented in Fig 5.

## Discussion

Soil nutrients, especially nitrogen are critical elements for plant growth and productivity. The bioavailability of nutrients in the soil solution may influence root growth, root proliferation and specific functional responses that depend on the prevailing nutrient status of the plant. Plasticity in root development can be seen in the way many plant species respond to an uneven distribution of nutrients (NO_3_^-^, NH_4_^+^ and decomposing organic matter) by ramification of the root system preferentially within nutrient-rich zones [31,32]. Nematode grazing can accelerate the mineralization of soil organic matter [33], increase the inorganic N available for uptake by plants [34], and also enhance plant root growth [35].

### High NH_4_^+^:NO_3_^-^ ratio results from stronger bacterial influence in the inoculation experiment compared with the exclusion experiment

There are striking differences in the NH_4_^+^:NO_3_^-^ ratio and the concentrations of inorganic N between the results of the two experiments. In the exclusion experiment, the concentration of NO_3_^-^ was higher than that of NH_4_^+^, as was the case in a previous experiment using the mesh exclusion technique to manipulate nematode numbers [6]; whereas in the inoculation experiment NH_4_^+^ was the dominant form of mineral N. This is probably a result of the sterilization of the soil in inoculation experiment which would favor the production of NH_4_^+^ and the removal of nitrifying bacteria which are slow to recolonize inoculated soil [36], and would thus result in reduced conversion of NH_4_^+^ to NO_3_^-^. The absolute concentrations of mineral-N in the inoculation experiment, were 80 μg g^-1^ NH_4_^+^ and 8 μg g^-1^ NO_3_^-^ (NH_4_^+^: NO_3_^-^ 10:1). This was at least double that of the exclusion experiment, with 10 μg g^-1^ NH_4_^+^ and 30 μg g^-1^ NO_3_^-^ (NH_4_^+^: NO_3_^-^ 0.3:1). In the exclusion experiment greater nematode abundance in the 1mm treatment were associated with initially higher soil NO_3_^-^ concentrations presumably because of increased mineralization, followed by an overall lower concentration, which was probably due to increased plant N uptake [13,35]. In contrast, the addition of nematodes in the inoculation experiment was not strongly associated with changes in NO_3_^-^ or NH_4_^+^, which were more strongly associated with changes in bacterial composition (Fig 5a–5c). The results are similar to those of Li [37], who found that excess NH_4_^+^ inhibited *Arabidopsis* root growth via a root tip mediated mechanism, however, we found no direct relationship between NH_4_^+^ and total root length. The effect of NH_4_^+^ on increased root diameter may be partially due to a direct effect in the elongation zone (*i*.*e*. cells stay short and wide, instead of elongating). Li also suggested that the inhibition mechanism of NH_4_^+^ on root growth was unlikely to be via an auxin based mechanism, and the direct effect of NH_4_^+^ inhibition was in the elongation zone [37]. Thus, in the inoculation experiment the high levels of NH_4_^+^ due to sterilization and bacterial activity may stimulate the production of root tips and thicker roots, while potentially repressing root elongation (Fig 5b and 5c). However, our data suggests that there may be a role for the GH3-2—IAA pathway in the NH_4_^+^ influence on root growth, with GH3-2 integrating the IAA and NH_4_^+^ influences.

### GH3-2 Integrates the IAA and NH_4_^+^ influence on root elongation

While both experiments showed evidence of an increase in IAA in the presence of greater faunal numbers, the IAA concentration was 10 times higher in the exclusion experiment than that in inoculation experiment. In the exclusion experiment, this resulted in a strong link between IAA concentration and root length. In both experiments, there was evidence that the plants perceived the increased level of IAA in the soil (*i*.*e*. increased relative GH3-2 expression compared to the internal UBQ5 standard). In addition in the inoculation experiment (high soil NH_4_^+^) there was evidence of NH_4_^+^:NO_3_^-^ interactions influencing GH3-2 expression however, in the exclusion experiment (low soil NH_4_^+^), no correlation was found between the NH_4_^+^ and NO_3_^-^ values and GH3-2 expression. As GH3-2 was measured relative to an internal standard (UBQ5), it was not possible to compare absolute expression levels across experiments. We hypothesize that the low NH_4_^+^:NO_3_^-^ ratio (0.3 in exclusion and 10 in inoculation) reduced the inhibition of root elongation and the significantly greater IAA concentration with greater nematode abundance, further stimulated root growth in the exclusion experiment (Fig 5a).

Zhang and Forde [32, 38] showed that a localized supply of NO_3_^-^ stimulated lateral root growth in *Arabidopsis* and this may not solely involve its role as an N source. In the exclusion experiment, NO_3_^-^ levels were greater than in the inoculation experiment and highest in the 5 μm treatment with smaller nematode populations. In contrast to Zhang and Forde [32], in the exclusion we found higher levels of NO_3_^-^ reduced the number of root tips generated. However, in combination with high levels of NH_4_^+^ in the inoculation experiment, NO_3_^-^ correlated with the production of GH3-2 which correlated with increased rates of root growth.

In conclusion, we found evidence that with greater bacterial-feeding nematode abundance, roots responded positively to increases in nitrogen and soil IAA content by an auxin-dependent pathway. While this was true with a low NH_4_^+^: NO_3_^-^ ratio, bacteria—nutrient interactions were the main drivers of root growth under high the NH_4_^+^ conditions.

## Supporting Information

S1 Fig The above pot was with nylon bags of 1mm pore diameter mesh, and the below pot was with nylon bags of 5 μm pore diameter mesh. (TIFF)
