# Supplementary figures and images for: Soil Nitrogen Status Modifies Rice Root Response to Nematode-Bacteria Interactions in the Rhizosphere
Source: PLoS One. 2016 Feb 3;11(2):e0148021. doi: 10.1371/journal.pone.0148021 (PMC4739600; doi:10.1371/journal.pone.0148021)

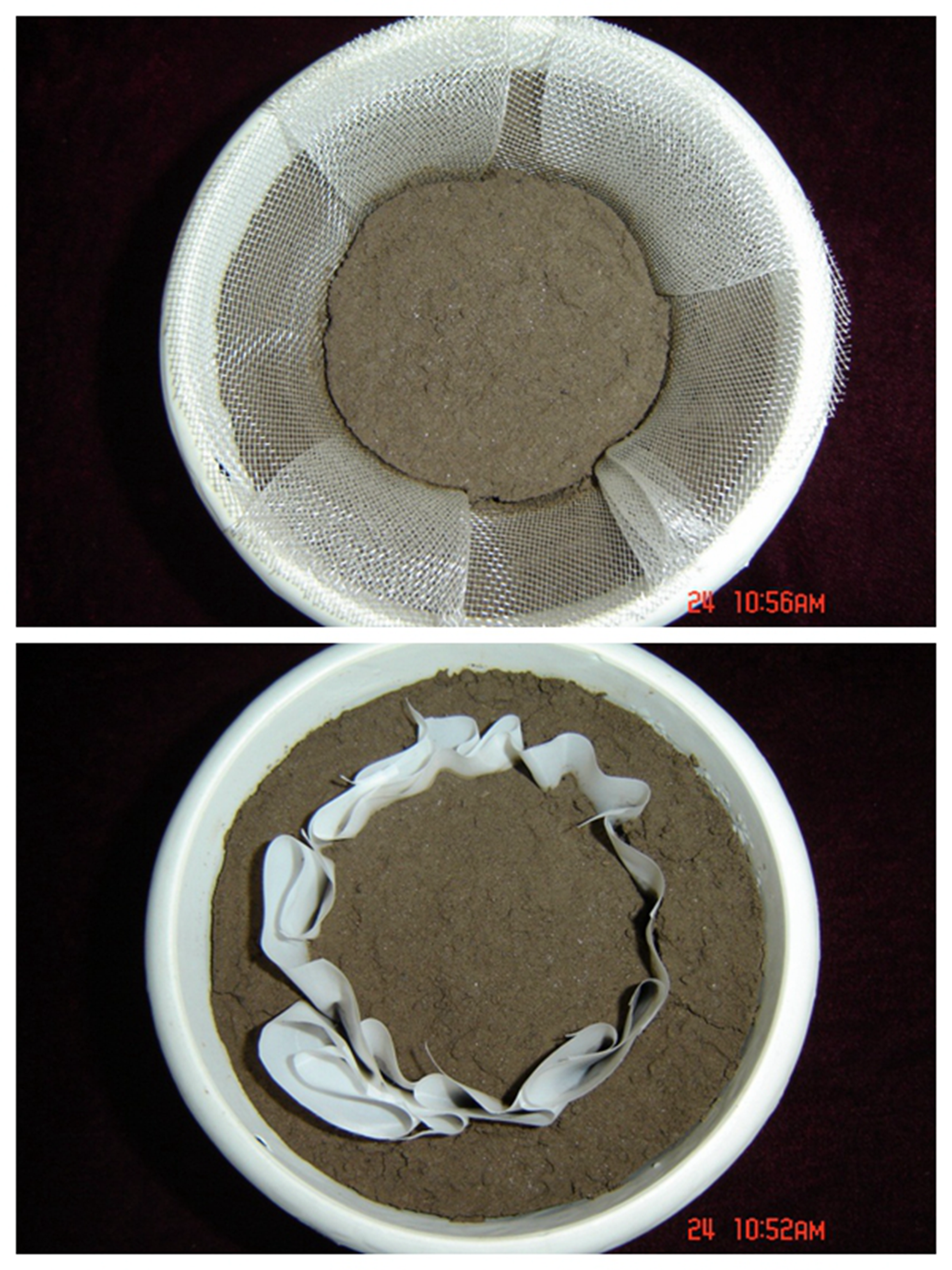

Supplement: S1 Fig — (TIFF) [file pone.0148021.s001.tiff]
